# Properties of epitaxial, (001)- and (110)-oriented (PbMg_1/3_Nb_2/3_O_3_)_2/3_-(PbTiO_3_)_1/3_ films on silicon described by polarization rotation

**DOI:** 10.1080/14686996.2016.1140306

**Published:** 2016-03-09

**Authors:** Muhammad Boota, Evert P. Houwman, Matthijn Dekkers, Minh D. Nguyen, Kurt H. Vergeer, Giulia Lanzara, Gertjan Koster, Guus Rijnders

**Affiliations:** ^a^ Faculty of Science and Technology, MESA+ Institute for Nanotechnology, University of Twente, P.O. Box 217, 7500AEEnschede, the Netherlands; ^b^ Engineering Department, University of Rome “ROMA TRE”, Via della Vasca Navale 79, 00146Rome, Italy; ^c^ SolMates BV, Drienerlolaan 5, Building 6, 7522NBEnschede, the Netherlands

**Keywords:** PMN-PT, pulsed laser deposition, orientation control, ferroelectricity, piezoelectricity, thin film, epitaxy

## Abstract

Epitaxial (PbMg_1/3_Nb_2/3_O_3_)_2/3_-(PbTiO_3_)_1/3_ (PMN-PT) films with different out-of-plane orientations were prepared using a CeO_2_/yttria stabilized ZrO_2_ bilayer buffer and symmetric SrRuO_3_ electrodes on silicon substrates by pulsed laser deposition. The orientation of the SrRuO_3_ bottom electrode, either (110) or (001), was controlled by the deposition conditions and the subsequent PMN-PT layer followed the orientation of the bottom electrode. The ferroelectric, dielectric and piezoelectric properties of the (SrRuO_3_/PMN-PT/SrRuO_3_) ferroelectric capacitors exhibit orientation dependence. The properties of the films are explained in terms of a model based on polarization rotation. At low applied fields domain switching dominates the polarization change. The model indicates that polarization rotation is easier in the (110) film, which is ascribed to a smaller effect of the clamping on the shearing of the pseudo-cubic unit cell compared to the (001) case.

## Graphical abstract

**Figure:**
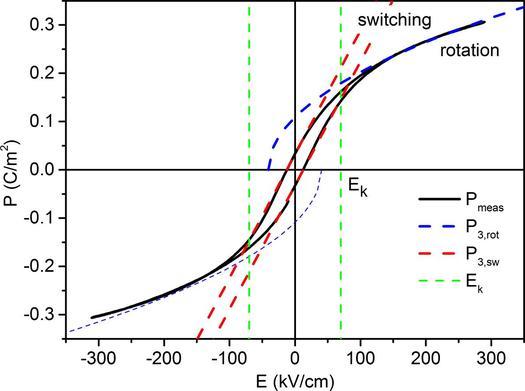

## 1. Introduction

Piezoelectric ceramic materials like PbZr_x_Ti_1-x_O_3_ (PZT) and (PbMg_1/3_Nb_2/3_O_3_)_1-x_-(PbTiO_3_)_x_ (PMN-PT) are used in a wide range of devices in both sensing and actuation applications, employing their piezoelectric property to directly convert mechanical energy into electrical energy or vice versa. There is a strong effort to fabricate epitaxial ferroelectric PZT and relaxor PMN-PT thin film based devices on silicon substrates for ferroelectric oxide thin film based electronic, photonic and MEMS (microelectromechanical systems) devices [1,2]. Bulk relaxor ferroelectrics like PMN-PT (x ≈ 0.33) show superior piezo-response, exhibiting 5–10 times larger piezoelectric coefficients than bulk PZT ceramics. This material has also a very large electromechanical coupling coefficient, k_33_ ≈ 0.9 [3,4]. Therefore it may also be an important material for thin film applications, necessitating the development of reliable thin film deposition techniques and the understanding of the properties of such thin films.

The growth of epitaxial PMN-PT films using appropriate buffers is challenging because of the large lattice mismatch between the film and the Si substrate. Another challenge is to control the crystallographic orientation of the PMN-PT films. The ferro- and piezoelectric properties of the most noticeable ferroelectric oxides (specifically the pure ferroelectric (PZT) and the relaxor ferroelectric PMN-PT) are strongly correlated with the crystallographic orientation of the thin films.

A number of heterostructures has been developed to achieve epitaxial growth of PMN-PT films on silicon. Baek et al. [4] reported the deposition of high crystalline quality, epitaxial PMN-PT films on a SrTiO_3_ buffered silicon substrate. Wang et al. [5] developed a SrTiO_3_/TiN/Si heterostructure, whereas Tsang et al. [6] demonstrated that the MgO/TiN/Si bilayer system can be successfully used to obtain epitaxial growth of PMN-PT on silicon. The yttria-stabilized zirconia/ceria (YSZ/CeO_2_) heterostructure is one of the most extensively investigated layer stacks for epitaxial growth of ferroelectrics oxide films on silicon [7,8]. There are reports on the deposition of (001)- and (110)-oriented epitaxial PMN-PT films using appropriate buffer layer(s) and/or electrode material.[4–10]. However, there is no literature about the control of the crystallographic orientation of epitaxial PMN-PT films on a silicon substrate with the same buffer layer(s) and/or electrode material. If one can ensure that film growth is the same in both orientations, while the intrinsic material properties in the film are kept the same, such a model system would allow one to compare the measured properties of these films and relate possible differences to the different crystal growth orientations only.

In this work, epitaxial PMN-PT films with (001) or (110) orientation are controllably fabricated on silicon substrates by pulsed laser deposition (PLD) using the *same* buffer layer/base electrode materials stack. The top monolayer of the base electrode is modified by choosing different deposition conditions (see details below) to realize a certain growth orientation. In this way stable PMN-PT films in the same substrate-induced in-plane strain state are realized and a clean model system is obtained to investigate and compare the effect of structure and crystallographic orientation on the ferro- and piezoelectric properties of epitaxial PMN-PT films on a silicon substrate.

We describe the ferroelectric, piezoelectric and dielectric properties of the films in terms of a model based on polarization rotation. At lower fields polarization switching dominates the film properties. Due to the different growth orientations (but equal in-plane strain state) we hypothesize that the polarization vector in our PMN-PT films rotates along different paths of the intrinsic energy landscape under the influence of the applied field, when scanning through polarization loops. The measurements indeed show that within the framework of the model the field dependence of the polarization angle depends on the growth orientation and thus the rotation path.

## 2. Experimental methods

**Figure 1.**
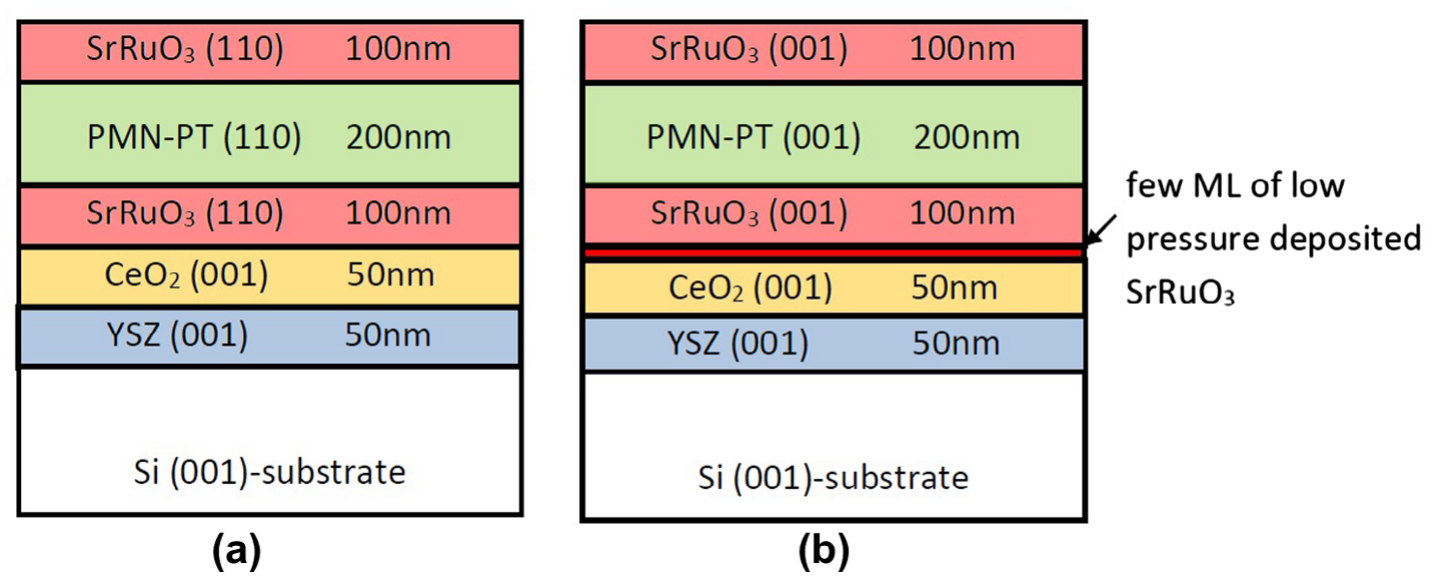
Schematic layer stack of (a) (110)-oriented and (b) (001)-oriented SRO/PMN-PT/SRO ferroelectric capacitors. ML stands for monolayer.

Two types of PMN-PT based capacitor structures were grown on a Si substrate, with either (001) or (110) growth orientation. The layer stack of the capacitor is shown schematically in Figure 1. The 200 nm thick epitaxial PMN-PT films were fabricated using 100 nm thick SrRuO_3_ (SRO) base and top electrodes. The SRO/PMN-PT/SRO structures are deposited on a (001)-oriented silicon substrate buffered with a CeO_2_(001)/YSZ(001) bilayer stack (each layer is 50 nm thick). All layers are deposited in series, without breaking the vacuum, by means of pulsed laser deposition (PLD) using a KrF Excimer laser (248 nm wavelength). The substrate was placed at a distance of 6 cm from the target. The PMN-PT films deposition was performed at a laser fluence of 2.25 J cm^–2^ and 4 Hz repetition rate.

In order to achieve epitaxial growth of the oxides on Si a buffer layer of YSZ is used, which was grown at a substrate temperature of 800°C. YSZ can grow heteroepitaxially on silicon as it scavenges the native Si oxide layer under specific deposition conditions, allowing the reproducible coherent growth of the oxides on a silicon substrate [11]. A following layer of ceria was grown at 800°C on the YSZ buffer layer with a cube-on-cube epitaxial relationship which can therefore be used as a second buffer layer to reduce the lattice mismatch between YSZ and the base electrode SRO layer. The in-plane mismatch between the fluorite structure of CeO_2_ and the perovskite pseudocubic SRO crystal is only 2.2% if the perovskite cubic unit cell of SRO is rotated by 45° with respect to the CeO_2_ unit cell [12,13]. To achieve the two desired orientations the first SRO monolayers can be deposited under either reduced oxygen pressure conditions (<10^−5^ mbar) and a high substrate temperature of 800°C leading to (001)-oriented growth followed by deposition of the main part of the SRO bottom electrode at 600°C, which leads to (001) growth of the SRO or skipping the low pressure/high temperature step resulting in (110)-oriented SRO [7,14,15]. The subsequent PMN-PT layer deposited at a substrate temperature of 600°C and an oxygen pressure of 0.28 mbar copies the orientation of the underlying SRO layer. The SRO top electrode was again deposited at 600°C. After deposition the samples were cooled down *in situ* from deposition temperature to room temperature under a 1 bar oxygen pressure.

The structural properties and epitaxial relationships were examined by X-ray diffraction (XRD; PANalytical X^1^pert PRO MRD, Almelo, the Netherlands). Ferroelectric capacitor devices, 200 × 200 μm^2^ in size, were patterned by photolithography and structured by argon ion beam milling. Ferroelectric hysteresis (*P* – *E*) loops were measured with a ferroelectric tester (AixACCT TF Analyser 3000, Aachen, Germany), using a bipolar triangular pulse at a frequency of 1 kHz and varying amplitude. Fatigue measurements were performed using a rectangular bipolar pulse train at a frequency of 10 kHz and with 150 kV cm^–1^ amplitude. The effective longitudinal piezoelectric coefficient (*d*_33_ – *E*) loops were measured with a laser Doppler vibrometer (Polytec MSA-400, Irvine, CA, USA), with a sinusoidal excitation of 8 kHz on top of a stepwise changing *DC* bias voltage using a lock-in technique. The relative dielectric constant versus electric field (*C* – *E*) measurements were performed with a Keithley 4200 (Tektronix, Beaverton, OR, USA) instrument at 10 kHz.

## 3. Experimental results

### 3.1. Structural analysis

**Figure 2.**
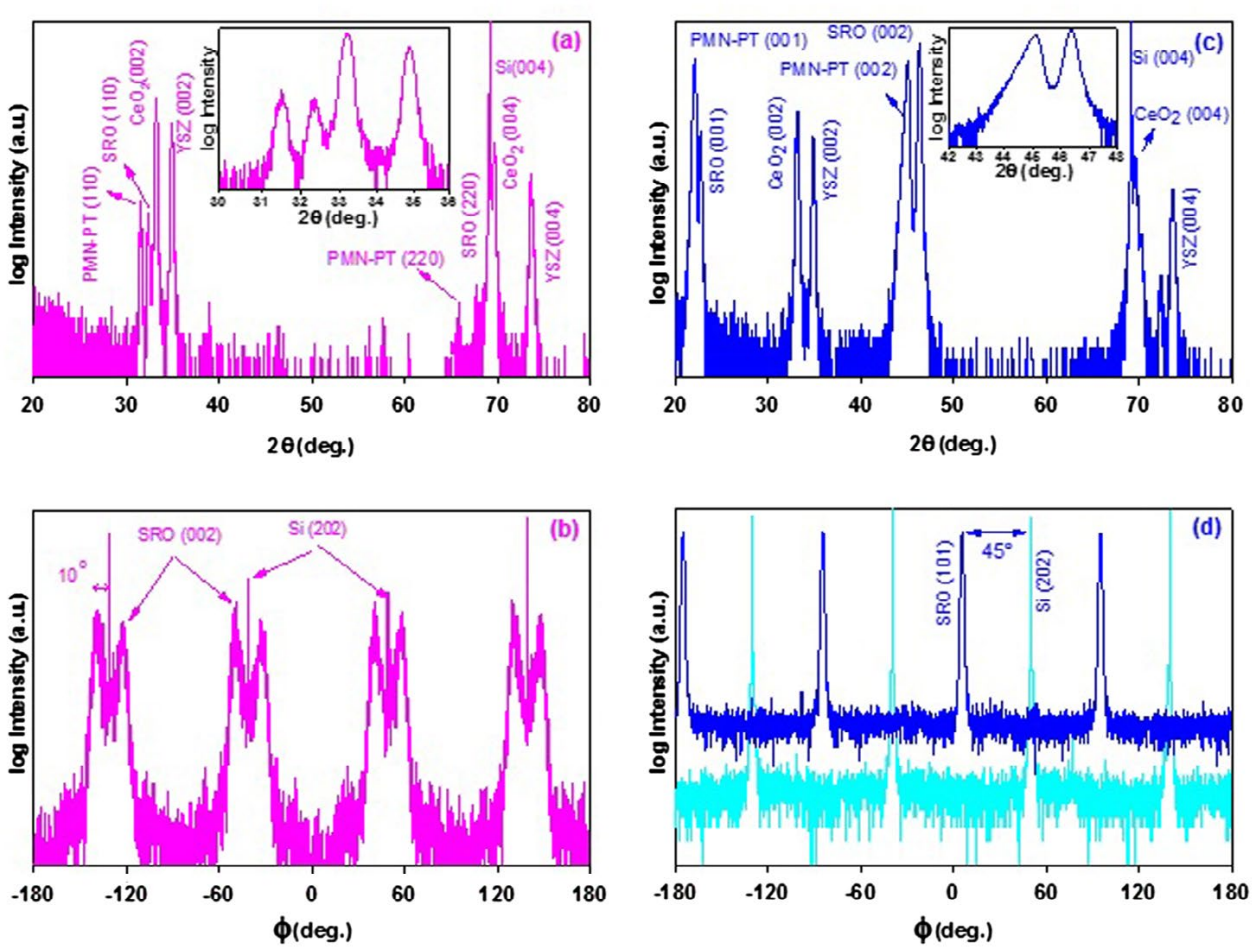
(a) XRD-2 diffractogram of 200 nm thick (110)-oriented PMN-PT film deposited on SRO(110)/CeO_2_(001)/YSZ(001) trilayer system on a silicon substrate. The inset shows an expansion of the (110) reflections. (b) Corresponding *φ*-scan of the Si substrate and SRO bottom electrode. (c) XRD pattern of 200 nm thick (001)-oriented PMN-PT film deposited on a SRO(001/CeO_2_(001)/YSZ(001) buffered Si substrate. The inset shows the (002) reflections. (d) Corresponding *φ*-scan of the Si substrate and SRO bottom electrode.

Figure 2(a) shows the *θ* – 2*θ* diffraction pattern of a PMN-PT film grown on the SRO/CeO_2_/YSZ heterostructure on silicon. The reflection peaks indicate that the PMN-PT film is in the perovskite phase and has a (110) orientation. No extra peaks that correspond to impurity phases or other orientations are observed. The FWHM of the rocking curve of the PMN-PT (110) peak is 1.6°. *φ*-scans of the CeO_2_/YSZ bilayer stack show four identical sets of peaks located at the same angles as the Si-substrate, separated by 90°, proving that CeO_2_(001)/YSZ(001) bilayer is grown with a relaxed epitaxial relationship with the Si(001) substrate [8,9]. The *φ*-scan in Figure 2(b) shows a peak doubling of the pseudocubic (222) direction of the 45° tilted SRO pseudocube peaks positioned at + 10° and –10° with respect to the position of the silicon (202) peak. This indicates the presence of twin domains in the film as was previously also observed by Hou et al. [16] for SRO deposited directly on YSZ/Si(001) and by Dekkers et al. [7] for SRO on CeO_2_/YSZ/Si(001). Because of the very small lattice mismatch between the SRO bottom electrode and the successive PMN-PT layer, the growth of PMN-PT follows the orientation of the bottom electrode. Therefore the twin domain structure also exists in the PMN-PT film, as was determined by *ϕ*-scan XRD analysis (not shown). The fairly large rocking curve width of the PMN-PT (110) peak is ascribed to the twin domain structure. The out-of-plane lattice constant of the PMN-PT (110) is obtained as 0.567 nm, which is slightly (–0.4%) less than that of an unstrained 45° tilted pseudocube (0.569 nm = √2*a*_*pc*_, with *a*_*pc*_ = 0.4022 nm), hence the PMN-PT (110) film is slightly tensile strained in the film plane. Figure 2(c) shows the *θ* – 2*θ* scan of the (001)-oriented PMN-PT films. Also here no additional peaks corresponding to impurity phases or other orientations were detected. The FWHM of the rocking curve of the PMN-PT (002) peak is 0.7°, which is half that of the (110)-oriented PMN-PT films. From the inset of Figure 2(c) we deduce that part of the PMN-PT (002) reflection is slightly shifted to lower angles from the angle expected for rhombohedral, unstrained PMN-PT. The broad shoulder is attributed to be due to a *compressive* strain of the in-plane lattice constant of the part of the layer in contact with the bottom SRO layer, relaxing to the bulk lattice parameter [17]. The maximum of the reflection corresponds to an out-of-plane lattice parameter of 0.4018 nm, indicating that the bulk of the film is slightly *tensile* strained, as is also expected from the differences in thermal expansion coefficients of film and substrate. Thus the compressive strain in the interface layer is expected to arise from cube-on-cube epitaxial growth of PMN-PT on the SRO with a much smaller lattice constant, while with increasing PMN-PT thickness the epitaxial strain is relaxed by defect incorporation.

### 3.2. Ferroelectric, dielectric and piezoelectric properties

**Figure 3.**
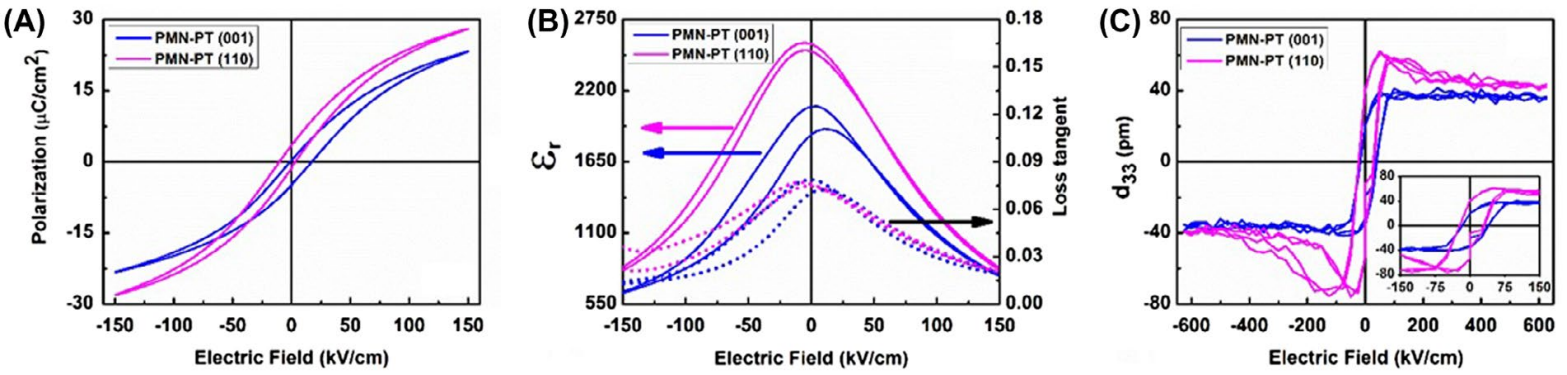
(a) Polarization hysteresis loop before fatigue treatment; (b) relative dielectric constant *ɛ*_*r*_ and dielectric loss tangent loops; (c) piezoelectric coefficient *d*_33_ of 200 nm thick (001)- and (110)-oriented PMN-PT films on CeO_2_/YSZ buffered Si(001) substrate, using SRO electrodes.

First, we note that the observed film properties are reproducible for different films (for each orientation several films were grown and measured), indicating that the observed differences are related to the growth orientation and not to variations in growth conditions. Typical ferroelectric hysteresis loops (*P* – *E*) of the PMN-PT films with different crystallographic orientation, measured before any fatigue treatment, are shown in Figure 3(a). The loops exhibit strong slanting and narrow hysteresis typical for bulk relaxors. The *P* – *E* loop of the (001) film shows a positive voltage shift, whereas that of the (110) films is slightly negatively shifted. Apart from the voltage shifts the loops appear to be symmetric. The polarization of the (110)-oriented film at high fields is significantly larger than that of the (001)-oriented film.

Somewhat naively one can interpret the difference in polarization values to be due to the different orientations of the pseudocubic [111]_*pc*_ direction, which is the preferential polarization direction in bulk PMN-PT. The length of the projection of this vector on the film normal direction is a factor $\sqrt{2/3}$ for a (110)-oriented film and $\sqrt{1/3}$ for a (001)-oriented film, respectively, of the length of the [111]_*pc*_ vector (see Figure 6). Thus one would expect in a first approximation that the ratio of the measured polarizations for the films with different growth orientations to be $P_{\left(110\right)}/P_{\left(001\right)}=\sqrt{2}$. Experimentally the ratio is about 1.2 at high fields. However if one assumes that the polarization can rotate freely in the relaxor material, this argument is not valid, since the polarization angle then depends on the strain state and applied field, as we will see below within the polarization rotation model.

Another difference between the loops is the relatively large shift of the loop of the (001) film towards positive field direction, indicating the existence of a positive self-bias *E*_*sb*_ ($E_{sb}=\left(E_{c}^{+}+E_{c}^{-}\right)/2$) of magnitude + 8.1 kV cm^–1^ ($E_{c}^{+}$ and $E_{c}^{-}$ are the coercive fields of the rising and falling branches of the *P* – *E* loop). The *P* – *E* loop of the (110)-oriented PMN-PT films shows a comparatively small, negative self-bias of –3.6 kV cm^–1^. Typically, in perovskite ferroelectrics a compressive in-plane stress results into a positive imprint, whereas in-plane tensile stress acts in the opposite way [18]. Note that the difference of thermal expansion coefficients between PMN-PT and the silicon substrate would suggest the presence of in-plane tensile strain in both films [19], which cannot therefore explain the difference in field shift for both cases. The XRD analysis suggests that the near bottom electrode part of the (001)-oriented PMN-PT film shows a strain gradient, with maximum strain at the base electrode/PMN-PT interface and relaxing into the film by the incorporation of lattice defects. It was previously shown that such a strain gradient can give rise to a large build-in electrical field, which is a possible cause for the self-bias voltage of this film [17]. The (110)-oriented film shows a smaller self-bias with opposite sign. In this film strain relaxation is much easier because of the high density of structural defects, due to the twinning of the crystal structure of the bottom electrode and PMN-PT film. This is consistent with the XRD rocking curve measurement showing more peak broadening, hence a larger tilt angle range of the crystallites within the film and therefore a higher density of structural defects as compared with the (001) films. The increased strain relaxation causes the strain gradient layer to be very thin, as is evidenced by the observation that the (110) reflection does not show an obvious asymmetry (shoulder). Also the self-bias voltage is low and even negative. The latter observation suggests that any build-in field – if present at all – is (over-) compensated by negatively charged defects in the film–substrate interface layer [17].

It is instructive to compare the *P* – *E* loops with those of (001)-poled single-crystal PMN-PT as presented in [27]. There the loops show no hysteresis, no self-bias, a linear field dependence in a very narrow field range between about –5 and 5 kV cm^–1^, associated with domain wall motion, and a gradual increase up to saturation outside this field range due to polarization rotation. For the films discussed here there is significant hysteresis, hence domain wall pinning; a finite field-bias; a 20-fold wider linear section of the loop, and a much slower increase of the polarization by rotation with increasing field. These effects are attributed to the clamping to the substrate.

The relative dielectric constant *ɛ*_*r*_ and dielectric loss tangent curves of the PMN-PT films are shown in Figure 3(b). A very small distance between the peaks of the two branches of the loop, much smaller than the width of the opening in the hysteresis loop, is observed. The (110)-oriented film has a larger dielectric constant than the (001)-oriented film over the full field range. The losses are approximately equal.

The loop of the measured effective piezoelectric coefficient *d*_33_ = ∂ *z*/ ∂ *V* of the (001)-oriented film (Figure 3(c)) is square, symmetric and saturates at about 39 pm V^–1^. The loop of the (110) film is asymmetric and shows strong maxima. These maxima occur at different negative applied fields, depending on the direction of the field change. The field range of the sloped part of both hysteresis loops is approximately as wide as that of the *P* – *E* loops, while the coercive fields of the *d*_33_ – *E* loops are significantly larger than of the *P* – *E* loops. The nearly constant *d*_33_ of the (001) film implies that the (average) unit cell height increases linearly with applied field, whereas the dimension of the unit cell in the [110] direction of the (110) film is more sensitive to the change in field at low fields than at higher field strengths. However *d*_33_ saturates at nearly the same value as for the (001) film. The maximum *d*_33_ values attained in these films are significantly smaller than the bulk values reported, up to 2800 pm V^–1^ [20]. The large effective *d*_33_ values reported for PMN-PT single domain, single crystals, poled in the (001) or (110) direction are ascribed to the large contribution of the large shear component $d_{15}^{R}$ of the rhombohedral phase to *d*_33_. However, in the films considered here, shearing is largely obstructed by the clamping to the substrate and the counteracting shear of adjacent grains with the polar axis in different in-plane directions.

### 3.3. Cycling stability of the ferroelectric response

**Figure 4.**
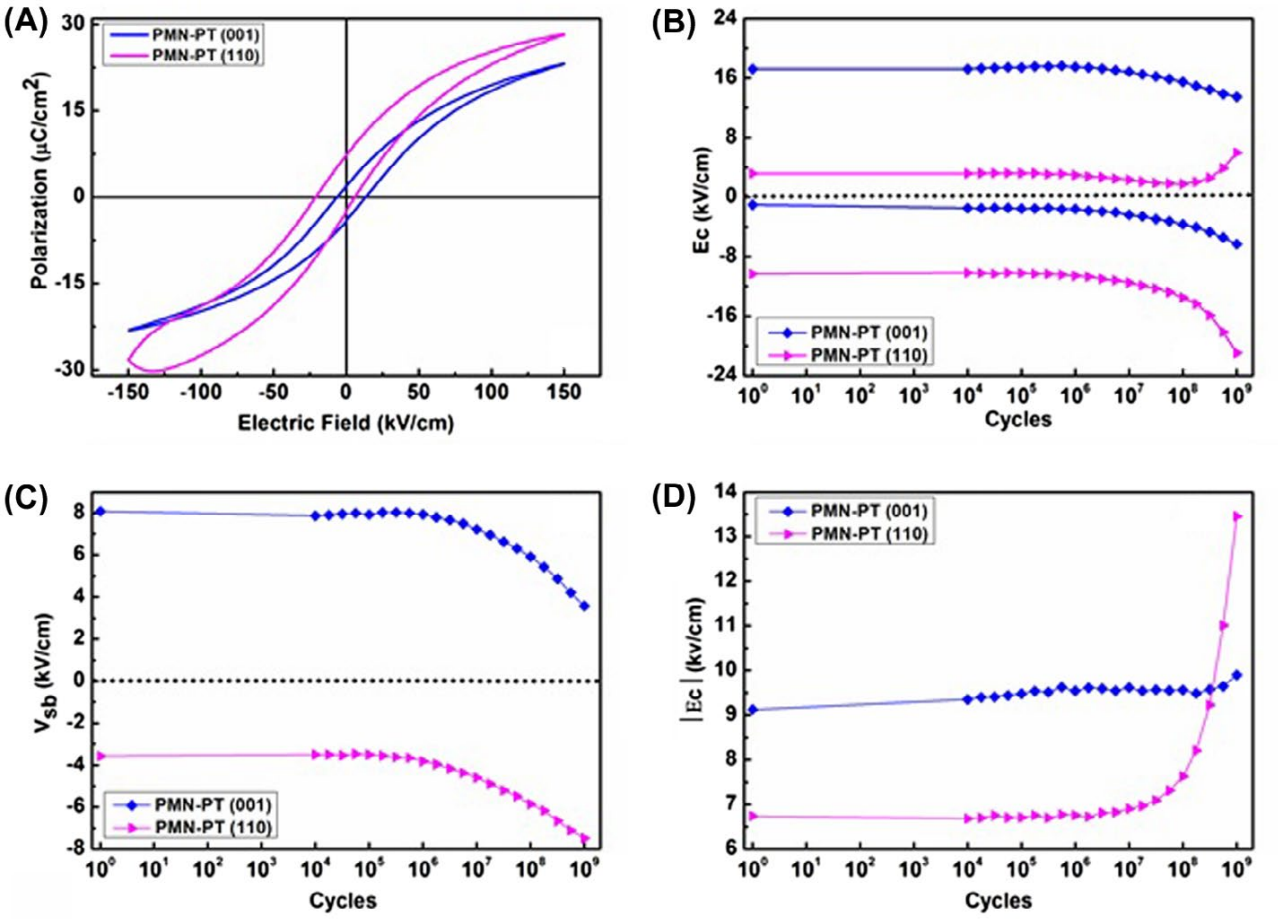
(a) *P* – *E* loops after fatigue treatment (10^9^ cycles); (b) coercive fields; (c) self-bias field; and (d) average coercive field versus number of ageing cycles performance of (001)- and (110)-oriented PMN-PT films on CeO_2_/YSZ buffered Si(001)-substrates, using SRO electrodes.

To analyse the effect of orientation and structure on the stability of the ferroelectric response, both films were subjected to a large number (10^9^) of switching cycles. The main fatigue feature of the (001)-oriented films is a small field shift of the *P* – *E* loop upon cycling, reflected in shifting coercive fields (Figure 4(b)) and thus a changing self-bias field (Figure 4(c)). On cycling the self-bias field, generally ascribed to an inhomogeneous fixed charge distribution in the piezoelectric, asymmetric electrodes or a thin insulating layer separating one of the electrodes and the piezoelectric, is compensated by a redistribution or compensation of the fixed charges through a slow field driven process. The average coercive field, hence the opening of the *P* – *E* loop, remains nearly constant upon cycling (Figure 4(d)). The *P* – *E* loop of this film (Figure 4(a)) after fatigue treatment is very similar to the initial loops, indicating that there is no increase of leakage currents. The (110)-oriented film also shows a slight shift along the field axis of the *P* – *E* loop (negative field direction) upon cycling up to about 10^8^ cycles, thus slight shifting of the coercive fields and a small change in self-bias field. However, above 10^8^ cycles the loop opening $\left|E_{c}\right|$ strongly increases and the remanent polarization doubles. Simultaneously the *P* – *E* loop of the (110) film shows strongly increased leakage currents. We interpret these changes as being due to an increased conductivity, especially of the grain boundaries of this film, due to the voltage cycling and the associated mechanical cycling via the piezo-electric effect. The (110) film is more sensitive to this fatigue mechanism, because the as-grown film has already more defective grain boundaries due to the crystallographic twinning.

## 4. Theory and applications

In relaxors the change in polarization is considered to be due to continuous rotation of the polarization vector (without significant extension of the polarization vector) and domain switching under influence of the applied electrical field [20,21]. In the following we develop a simple analytic approach based on these assumptions. This allows us to derive equations from the experimental data that capture the essential field dependence of the material properties and connects quantitatively the measured polarization, dielectric and piezo-electric hysteresis loops. This makes it possible to separate intrinsic effects from extrinsic effects, such as domain wall motion.

In an unstrained (001)-oriented film of PMN-PT material with rhombohedral symmetry the polarization can be oriented in the eight equivalent <111> directions, thus one expects the material to be divided in polarization domains with these eight polarization directions. At high enough field in the out-of-plane direction only the four domains with a polarization component in the direction of the applied field remain (in bulk this correspond to the ‘4R’ domain structure and the ‘4M_C_’ structure at high fields) and for very large fields, when the polarization in all domains is parallel to the field the symmetry becomes tetragonal and the system is in a single domain state. In principle all the properties of the film can be derived by numerical minimization of the average free energy of the clamped ferroelectric film, $F=\overset{}{\tilde{G}}-E_{3}P_{3}=G_{0}+\sum_{i}S_{i}\sigma_{i}-E_{3}P_{3}$, if the values of all stiffness and electrostrictive coefficients and elastic compliance were known (for example as done in [22]). $\overset{}{\tilde{G}}$ is the zero field energy of the clamped film. *G*_0_ is the standard Gibbs energy used in the thermodynamic theory of bulk ferroelectric crystals including elastic energy [23], *S*_*i*_ and *σ*_*i*_ are the components of the strain and stress vectors in Voigt notation, *E*_3_ is the applied field in the out-of-plane direction and *P*_3_ the polarization component in this direction. The model neglects the small energy contribution from the domain walls. The averaging is over the different domains. The unit cells in the four different domains have the same rhombohedral symmetry, therefore we assume that unit cells in each domain can be described with the same energy equation and thus that we may consider the film to be in a (quasi-) single domain state. We will use some results of the single domain model to obtain expressions for the field dependence of the film properties.

First we consider the (001)-oriented film, with in-plane 1- and 2-directions and the out-of-plane 3-direction. The direction indices also refer to the principle axes of the (001)-oriented pseudocube. In each of the four polarized domains the out-of-plane component of the polarization, ${\text{P}}_{3}^{\text{rot}}$, and the in-plane component, ${\text{P}}_{1,2}^{\text{rot}}$ can be written as:

(1) $$P_{3}^{\text{rot}}=P_{0}\sin\psi \;P_{12}^{rot}=\sqrt{P_{1}^{2}+P_{2}^{2}}=P_{0}\text{cos}\psi$$

where *P*_0_ is the vector length and *ψ* the polarization angle with the film plane. For a clamped single domain film the strain in the out-of-plane direction of a (001)-oriented pseudo-cube is in general given by [24]:

(2) $$S_{3}=aS_{m}+b\left(P_{1}^{2}+P_{2}^{2}\right)+cP_{3}^{2}=aS_{m}+bP_{0}^{2}+P_{0}^{2}\left(c-b\right)sin^{2}\psi$$

Here S_m is the misfit strain between film and substrate, $a=2s_{13}/\left(s_{11}+s_{12}\right)$, $b=Q_{12}-\left(a/2\right)\left(Q_{11}+Q_{12}\right)$, and *c* = *Q*_11_ – *aQ*_12_, with *Q*_*ij*_ and *s*_*ij*_ the electrostrictive and elastic compliances [25–27]. The right-hand side of Equation (2) is obtained after substitution of Equation (1). The values of all the parameters depend on the orientation of the unit cell. The shear of the clamped unit cell is [24]:

(3) $$S_{4}=Q_{44}P_{2}P_{3}=\frac{Q_{44}P_{0}^{2}\sin2\psi }{2\sqrt{2}}=S_{5}=Q_{44}P_{1}P_{3}$$

The piezoelectric coefficient of the film $d_{33}$ is obtained as:

(4) $$d_{33}=\frac{\partial S_{3}}{\partial E_{3}}=P_{0}^{2}\left(c-b\right)\frac{\partial \psi }{\partial E_{3}}\text{sin}2\psi +2P_{0}\frac{\partial P_{0}}{\partial E_{3}}\left[b+\left(c-b\right)sin^{2}\psi \right]=d_{33}^{\text{rot}}+d_{33}^{\text{ext}}$$

The first right hand term describes the effect of polarization rotation (index ‘rot’) on the piezoelectric coefficient and the second term that of polarization extension (‘ext’). At very high voltages, when *ψ* ≈ 90°, the unit cell is practically tetragonal (*S*_4_ = *S*_5_ = 0) with the polarization in the 3-direction, $\partial \psi /\partial E_{3}\approx 0$. Thus Equation (2) reduces to $S_{3}=aS_{m}+cP_{3}^{2}$ and Equation (4) to $d_{33}=d_{33}^{\text{ext}}=2cP_{0}\frac{\partial P_{0}}{\partial E_{3}}$ and we can identify an effective electrostrictive constant for the clamped film at high fields, $Q_{33}^{\text{eff}}=c$. This corresponds to the usual formula $d_{33}=d_{33}^{ext}=d_{33}^{\prime }-2s_{13}d_{31}^{\prime }/\left(s_{11}+s_{12}\right)$ for a clamped film, where we have defined $d_{33}^{\prime }=2Q_{11}\varepsilon_{0}\varepsilon_{33}P_{0}$ and $d_{31}^{\prime }=2Q_{12}\varepsilon_{0}\varepsilon_{33}P_{0}$. Although the latter expressions have the same structure as that of the longitudinal and transverse piezoelectric coefficients of a free crystal of PMN-PT, it is only a formal similarity, but the parameter value of *ɛ*_33_ that enters these relations is still determined by the clamping. With the literature values for the *Q*_*ij*_ and *s*_*ij*_ parameters of the pseudocubic phase we obtain c = 0.0137.

**Figure 5.**
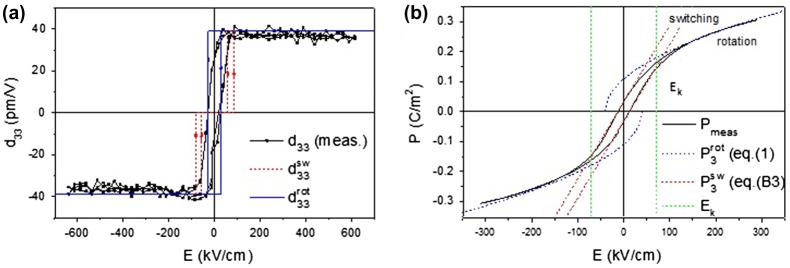
(a) *d*_33_ – *E* hysteresis loop of (001)-oriented PMN-PT film, fitted with the rotation and switching model curves. (b) Polarization hysteresis loop, fitted with polarization rotation at high fields and switching model at low fields. (c) Polarization angle and shear angle versus applied field.

**Figure A1.**
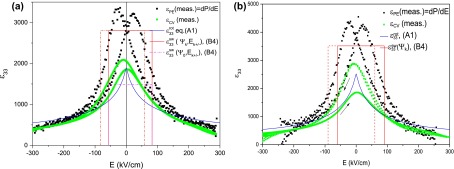
Measured relative dielectric constants versus field obtained from differentiation of the P – E loop ($\varepsilon_{PE}\left(meas\right)$) and from C – V measurements ($\varepsilon_{CV}\left(meas\right)$). The calculated curves follow from the model for the rotation ($\varepsilon_{33}^{rot}$) and switching ($\varepsilon_{33}^{sw}$) branches of the P – E loop. (a) (001)-oriented PMN-PT device. (b) (110)-oriented device.

Measurements (Figure A1 in Appendix A) show that the high field values of *ɛ*_*r*_ (which should be equal to *ɛ*_33_ in the theoretical formulas) saturate at about 250 at 300 kV cm^–1^ (for larger fields the *ɛ*_*r*_ determined from *C* – *V* measurements is strongly influenced by leakage currents). With these values the (high field) piezoelectric constant is found to be *d*_33_ = 42 pm V^–1^, very close to the measured value of *d*_33_ ≈ 39 pm V^–1^ (Figure 5(a)). Thus the experimental high field value is well predicted by the model for a clamped, (001)-oriented, tetragonal film, suggesting that at the maximum field values used in the *d*_33_ – *E* measurements *d*_33_ is determined by the polarization extension mechanism. At lower voltages the rotation dependent term in Equation (4) determines the piezo-electric coefficient, because $\partial P_{0}/\partial E_{3}$ is small,

(5) $$d_{33}^{rot}=P_{0}^{2}\left(c-b\right)\frac{\partial \psi }{\partial E_{3}}sin2\psi$$

This is the central relation from which we establish a link between the experiments and the semi-empirical model described in the following. One expects a smooth transition from the rotation to the extension mechanism and thus a constant or smoothly changing *d*_33_ – *E* loop in the voltage range where the rotation mechanism fades out and the extension mechanism comes up. If the high field value of *d*_33_ is determined by extension, as the close match between theoretical and experimental values suggests, then the transition voltage range is below the maximum field applied in the *d*_33_ – *E* measurements (about 650 kV cm^–1^). Experimentally *d*_33_ is approximately constant over a wide voltage range and equal to *d*_33_ ≈ 39 pm V^–1^. Assuming that at relatively low voltages the measured polarization change is dominated by polarization rotation, the product of the angle dependent terms in ${\text{d}}_{33}^{rot}$ take the constant value *k*_1_

(6) $$\frac{\partial \psi }{\partial E_{3}}sin2\psi =k_{1}=\frac{d_{33}}{P_{0}^{2}\left(c-b\right)}$$

Through the term $\left(c-b\right)=\left(1+a/2\right)\left(Q_{11}-Q_{12}\right)$ the parameter *k*_1_ (units cm kV^–1^) is a sensitive function of the material parameters, especially of *s*_13_. It appears that a change of for example *s*_13_ by as little as 15% can change $\left(c-b\right)$ by a factor of 3. Because of this sensitivity we will not use the calculated value, but extract the value *k*_1_ from the polarization hysteresis measurements instead. Integration of Equation (6) gives an expression for the field dependence of the angle *ψ* of the polarization vector in the (001)-oriented unit cell:

(7) $$-2\left(k_{2}+k_{1}E_{3}\right)=cos2\;\psi$$

The (dimensionless) integration constant *k*_2_ defines the angle $\psi_{0}=0.5\text{acos}\;\left(-2k_{2}\right)$ at zero field. If the (001) (respectively (110)) oriented unit cell is unstrained the integration constant can be obtained from the assumption that at zero applied field the polarization vector is oriented in the body diagonal direction, at angle $\psi_{0}^{001}=atan\left(1/\sqrt{2}\right)$ = 35.3° ($\psi_{0}^{110}=54.7^{\circ }$). From this follows $k_{2}^{001}=-0.167$ ($k_{2}^{110}=0.167$) and a remanent polarization $P_{r}^{001}=P_{0}sin\psi_{0}$ =0.25 C/m^2^ ($P_{r}^{110}=$ 0.35 C/m^2^), using *P*_0_ ≈ 0.43 C m^–2^ [27]. However if the lattice is strained the polarization vector is tilted towards the film plane for tensile stress, resulting in a lower zero field angle *ψ*_0_ and consequently lower *P*_*r*_.

In short, assuming that polarization rotation dominates the *d*_33_ – *E* loop at lower voltages described by Equation (5), the measurements imply Equation (6). This results in the field dependent polarization angle defined by Equation (7). The coefficients *k*_1_ and *k*_2_ are obtained by fitting the polarization loop with Equations (1) and (7), from which a value for the material parameter ${\left(c-b\right)}_{001}$ is obtained. The parameter *c* follows from the high-field section of the *d*_33_ – *E* measurement where polarization extension dominates the piezoelectric effect. Differences in the *k* coefficients for (001)- and (110)-oriented clamped films then reflect the effect of the orientation of the film on the properties. Finally, we note that contrary to the *P* – *E* and *C* – *V* measurements the *d*_33_ – *E* measurements can be taken up to much higher field values, far into the regime where field dependent leakage currents start to dominate the electrical measurements. This is because the leakage currents do not influence the mechanical deformation of the film as long as the field can be applied. This allows the determination of the angle dependence of the polarization vector over a much larger field range than from electrical measurements.

Figure 5(b) shows the measured *P* – *E* loop of Figure 3(a) of the (001)-oriented device, corrected for a small constant leakage resistance and field-shifted over the small self-bias field. This is the largest field range in which the leakage resistance is low and constant. (For larger applied fields the leakage strongly increases, distorting the loop significantly.) The loop is fitted in the high field regions with Equation (1), using the field dependence of the polarization angle given by Equation (7), with $k_{1}^{001}$ and $\psi_{1}^{001}$ as fitting parameters. In the interval *E* = [–100, 100] kV cm^–1^ the hysteresis loop deviates strongly from the polarization rotation model. This is because we expect that in this field range polarization switching (the out-of-plane polarization component switches direction) is the dominant mechanism for polarization change. The *P* – *E* loop branches due to switching are indicated by the red dashed lines in Figure 5(b). (In Appendix B this is discussed further and the consequences for the device properties in this field range are described.) The fit of the polarization rotation model to the experimental loop is very sensitive to the values of these parameters, which can therefore be determined accurately ($k_{1}^{001}$ within 0.05 × 10^−8^ and $\psi_{1}^{001}$ within 2°). *k*_1_ determines the slope and curvature of the loop, while *ψ*_0_ sets the *P*-axis crossing. Figure 7(c) shows the polarization angle of the falling branch of the hysteresis loop as function of field for the fit parameter values $k_{2}^{001}=-0.45$ (*ψ*_0_=12.4°) and $k_{1}^{001}=$1.67x10^−8^ (m V^–1^). This corresponds to a value ${\left(c-b\right)}^{001}$=0.0126 (m^4^/C^2^), which can theoretically be obtained using the literature values for *Q*_*i*_ and *s*_*ij*_, but with $s_{13}=0.87s_{13}^{lit}$. The model suggests that the polarization vector tilt angles varies approximately linearly over a wide field range, [0, 500] kV cm^–1^. The angle rapidly drops to zero close to a critical value $E_{3}=E_{3\text{min}}=-\left(0.5+k_{2}^{001}\right)/k_{1}^{001}$ = −27.4 kV cm^–1^ where the polarization is lying in the film plane. For $E_{3}>E_{3\text{max}}=\left(0.5-k_{2}^{001}\right)/k_{1}^{001}$ = 570 kV cm ^– 1^ the polarization is normal to the film plane. This corresponds to the high field range of the *d*_33_ – *E* measurements, where *ψ* saturates at 90° and polarization extension determines the piezoelectric coefficient. Thus *d*_33_ is determined by polarization extension only above *E*_3max_. This corroborates the close match between the experimental high field value of *d*_33_ and the value calculated from the extension mechanism. Figure 7(c) also shows the calculated shear angle *S*_4_ = *S*_5_ of the rhombohedrally deformed pseudocube as function of the field, as calculated from Equation (3). At *E*_3_ = *E*_3*min*_ the shear angle is zero, indicating that the pseudocube has been tetragonally deformed. At zero field the shear angle is about 0.16° and increases with increasing field to reach a maximum of 0.38° at a field strength of about 275 kV cm ^– 1^. For larger fields the rhombohedral deformation reduces again and the pseudocube becomes more tetragonal. In principle one might be able to determine the shear angles from XRD data. However, the XRD reflections are not very sharp, probably because of the overlapping of the reflections arising from the four-fold symmetry of the crystallographic domain structure.

We started with the conjecture that the angle dependence of the polarization can be derived from the constant *d*_33_ at higher field, using Equation (5). This equation can be rewritten as $d_{33}=2Q_{33}^{\text{eff}}\varepsilon_{33}P_{3}$, with $Q_{33}^{\text{eff}}={\left(c-b\right)}^{001}$. The constant measured value of *d*_33_ for the (001) film seems fortuitous, since, according to the polarization rotation model, it arises from the cancellation of the angle dependences in the product *ɛ*_33_(*E*)*P*_3_(*E*) in Equation (5) for all field values, which is a consequence of the angle-field dependence in Equation (7). The fitting results in a zero field polarization angle of about $\psi_{0}^{001}=$ 12.4°, much less than of the bulk body diagonal direction. Together with the small observed tensile strain in the film (*S*_*m*_ = 0.0013 calculated from the difference in thermal expansion coefficients from film and substrate) this implies that the polarization easy axis direction is very easily changed under the influence of strain, $\psi_{0}^{001}\left(S_{m}\right)\approx \psi_{0}^{001}\left(0\right)+\left(\partial \psi_{0}^{001}/\partial S_{m}\right)S_{m}$, with $\psi_{0}^{001}\left(0\right)$ = 35.3° and $\partial \psi_{0}^{001}/\partial S_{m}$ equal to –17.6° per 10^−3^ in plane strain.

**Figure 6.**
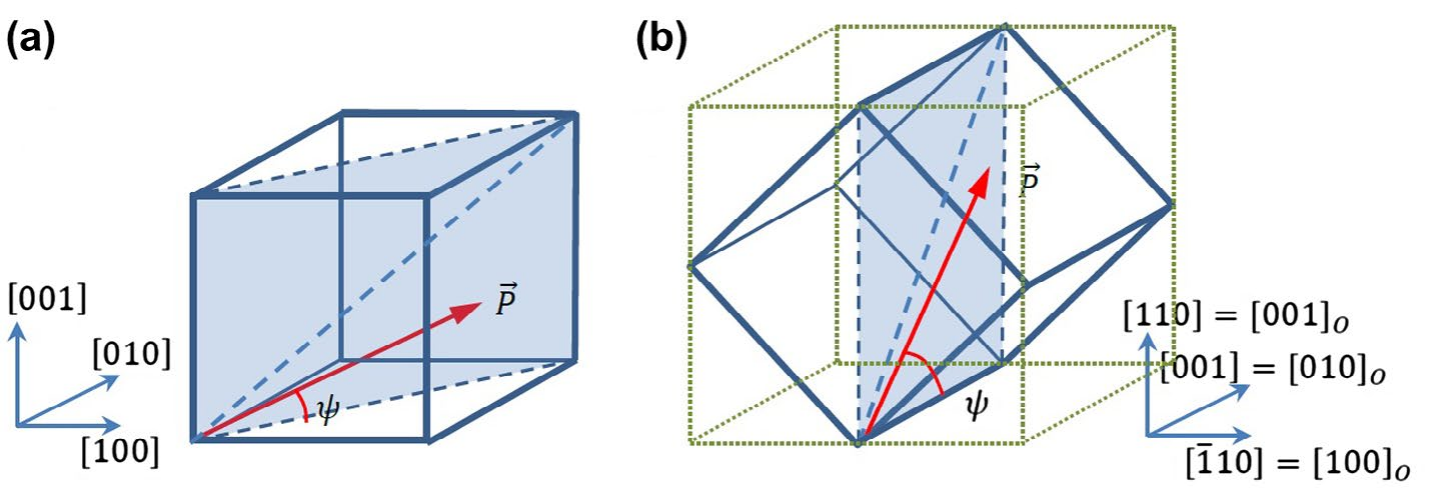
Rotation of the polarization vector in the $\left(\overset{}{\overset{¯}{1}}10\right)$ plane of (a) the (001)-oriented pseudocubic unit cell under the influence of the applied field E_3_; (b) the (110)-oriented unit cell. The green frame gives the orthorhombic unit cell.

In the case of the (110)-grown film a similar analysis as for the (001)-oriented film can be followed. In this case the film is not polarized in the [001] direction, but in the [110] direction. For the (001)- and (110)-oriented films the polarization vector rotates in opposite directions in the pseudocubic unit cell under the influence of the applied field (see figure 6). In the case of an unstrained (001)-oriented unit cell, the tilt angle with the film plane increases from $\psi_{0}^{001}=$ 35.3° rotating the polarization vector away from the in-plane [110] towards the out-of-plane [001] direction. For the tensile strained film discussed above the zero-field easy axis angle is found to be strongly reduced and equals 12.4°. In the unstrained (110)-oriented unit cell $\psi_{0}^{110}=$ 54.7° and the polarization rotates in the opposite direction as for the (001) film. One may therefore expect a different dependence of the tilt angle on applied field. Specifically the values of the coefficients *a*, *b* and *c* in Equation (2) are different for the (110)-oriented, clamped pseudo-cube, but the describing expressions for *d*_33_, *P*_3_ and *ɛ*_33_ have the same functional form when considering the orthorhombic representation. (The 45° tilted pseudocube can be described in terms of an orthorhombic unit cell (${\left[100\right]}_{O}\times {\left[010\right]}_{O}\times {\left[001\right]}_{O}$ with approximate dimensions $\sqrt{2}a_{pc}\times a_{pc}\times \sqrt{2}a_{pc}$).) Defining *P*_3_ in the [110] direction and *P*_1_ in the [001] direction, then *P*_2_ in the in-plane [1–10] direction is zero. It follows that *S*_4_ = 0 (no shear in the (101) plane), but *S*_5_ is a function of the polarization angle. Thus in the orthorhombic presentation the unit cell is slightly sheared in the short axis in-plane direction.

**Figure 7.**
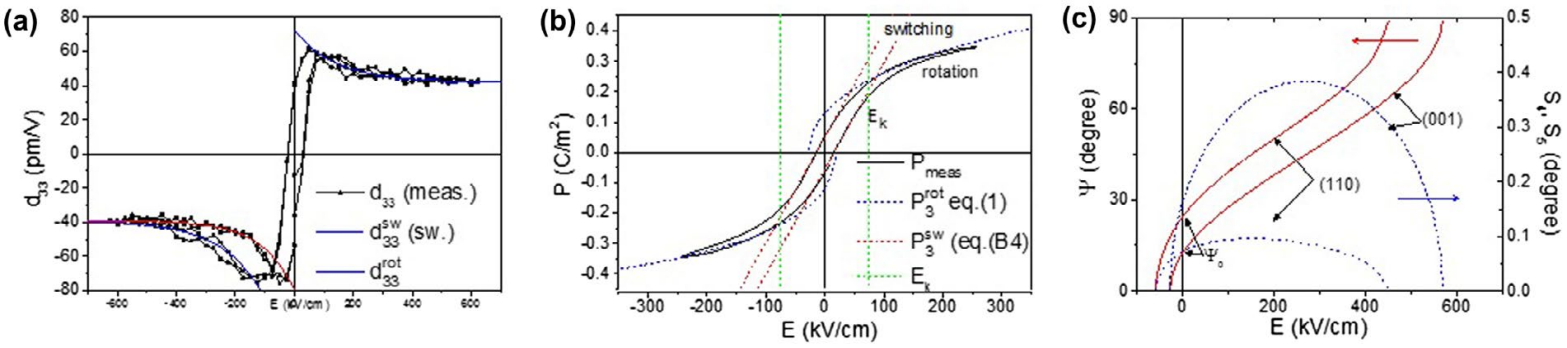
(a) *d*_33_ – *E* of (110)-oriented PMN-PT film fitted with the rotation model. (b) Polarization hysteresis loop, fitted with polarization rotation at high fields and switching model at low fields. (c) Polarization angle and shear angle versus applied field of the (001) and (110) films. The shear of the (110) film is (somewhat arbitrarily) a factor of 4 smaller than that of the (001) film, reflecting a weaker coupling between shear strain and polarization.

At high fields the measured value *d*_33_ becomes constant and approximately equal to that of the (001) film, implying that the products $P_{0}^{2}\left(c-b\right)\frac{\partial \psi }{\partial E_{3}}sin2\psi$ are equal for both orientations. The factors ${\left(c-b\right)}^{001}$ and ${\left(c-b\right)}^{110}$ are constants, thus the larger *d*_33_ value of the (110) film at lower field values, as compared to the high field values, indicates that the product $d_{33}∼{\left(\frac{\partial \psi }{\partial E_{3}}sin2\psi \right)}^{110}$ is now field dependent at low fields. This field dependence may be approximated by the functional form $d_{33}\approx \left[P_{0}^{2}{\left(c-b\right)}_{110}\right]\left[k_{1}^{110}+k_{3}^{110}\text{exp}\left(-k_{4}^{110}E\right)\right]$. From fitting to the *P* – *E* and *d*_33_ – *E* loops the *k*_*i*_ parameters are again determined and the field dependence of the polarization angle is obtained, as shown in Figure 7(c). We find $k_{1}^{110}$ = 1.84 x 10^−8^, corresponding to ${\left(c-b\right)}^{110}$ = 0.0123. $k_{2}^{110}$ = -0.22 m V^–1^ giving $\psi_{0}^{110}=$ 17.7°. $k_{3}^{110}$ and $k_{4}^{110}$ follow from the fit to *d*_33_ – *E)*. The different functional dependence of *ψ*(*E*) does not cause noticeable qualitative differences in the calculated *P* – *E* (and *C* – *V*) loops. Indeed the measured loops (see Figures 7(a) and (A1(b)) are equally well described by the modelled curves as in the (001) case. We note that the asymmetry of the *d*_33_ – *E* curves of the (110) film (broad peak of the falling branch as compared to the smaller peak of the rising branch at negative field bias and secondly high peaks at negative bias as compared to positive bias) also indicates that *ψ*(*E*) depends on the direction of the field change, especially for polarization down (negative bias) and thus on the applied field history. The large fields applied in the *d*_33_ – *E* loop giving rise to the asymmetry, could not be used for the *P* – *E* and *C* – *V* measurements. Therefore we have used the *ψ*(*E*) dependence derived from the positive field branch of $d_{33}$, for which the rising and falling curves are approximately equal, to calculate the curves in Figure 7(a), (c) and (d). Figure 7(b) shows again the good fit obtained with the rotation model. At high voltages the fitted rotation curves deviate somewhat from the measured loop. This is possibly due to the increased leakage current at these voltages in the (110) device.

The derived field dependence of the polarization angle (Figure 7(c)) in the positive field branch is steeper than for the (001) film, thus it appears that in the clamped film the polarization rotates somewhat easier in the (110) film towards the [110] direction than in the (001) film towards the opposite [001] direction under the influence of the applied field. The easier polarization rotation suggests also that there is less lattice distortion than in the (001) case. This implies a weaker coupling between the polarization components and the shear strain, thus a smaller value of $Q_{44}^{110}$ (in the orthorhombic coordinate frame) than $Q_{44}^{001}$. We think that the difference in *Q*_44_ values between the (001) and (110) grown films, which couples the polarization rotation to the shear of the crystal, may be an important reason for the differences in properties, which are associated with the more or less easier rotation of the polarization vector under the influence of the applied field. In the (110) sample with less induced shear, the shear of domains with different in-plane polarization orientations counteracts the shearing of neighbouring domains less than in the (001) film. Thus with increasing field the unit cell can more easily adapt its shape to the rotated polarization angle or, vice versa, if the unit cell cannot shear easily the polarization cannot rotate that much.

We conclude that the simple rotation model quantitatively couples the measured field dependence of polarization and piezoelectric coefficient fairly well, assuming coherent rotation of the polarization as the dominant mechanism for polarization change in the voltage range in which the polarization loops are measured. This is plausible for a good epitaxial film with homogeneous properties and strain state. In Appendix A we discuss consequences of the rotation model for the film permittivity and find reasonable correspondence between the model and experiment. As mentioned in the introduction at applied fields below the value *E*_*k*_, polarization switching dominates the polarization loop. In Appendix B a phenomenological model is developed to describe this behaviour and its consequences for the *C* – *V* and *d*_33_ – *E* loops.

## 5. Conclusions

In conclusion, relaxor PMN-PT thin films with (001) or (110) orientation were epitaxially grown on Si substrates with a SRO/CeO_2_/YSZ buffer layer using PLD. The PMN-PT films are phase pure and relax to bulk lattice parameters over a thin layer. The (110)-oriented films show larger polarization and dielectric permittivity values. The effective longitudinal piezoelectric coefficients *d*_33_ of both types of film approach the same value for large bias fields, about 39 pm V^–1^, significantly smaller than that of bulk PMN-PT. This is attributed to clamping by the substrate. The (001)-oriented film shows a relatively large self-bias voltage, which is ascribed to a strain gradient layer at the interface with the bottom electrode. On long term cycling the self-bias is slightly reduced, probably due to the introduction of charged defects in this strained layer. In the case of the (110)-oriented film the self-bias is small and has opposite sign and increases further on cycling.

The ferroelectric, dielectric and piezoelectric properties are described by a model, assuming that polarization rotation is the dominant mechanism for polarization change in the high electric field range. The rotation rate is larger for the (110)-oriented film than for the (001)-oriented film, which results in enhanced ferroelectric and dielectric properties, as is also observed experimentally. It is suggested that the difference in the value of the shear electrostriction parameter *Q*_44_ may play an important role in this. At low applied fields polarization change is dominated by polarization switching and domain wall motion. However, also at low fields the polarization rotation process appears to determine largely the dielectric properties.

The developed model makes it possible to disentangle different mechanisms for polarization change in clamped thin relaxor films (rotation, switching and extension), which dominate in different applied field regimes, and their effects on ferroelectric, dielectric and piezoelectric properties.

## Disclosure statement

No potential conflict of interest was reported by the authors.

## Appendix A – dielectric constant due to polarization rotation

The relative dielectric constant in the rotation part of the hysteresis loop is obtained from Equation (1) as:

(A1) $$\varepsilon_{33}^{rot}\left(E\right)=\frac{1}{\varepsilon_{0}}\frac{\partial P_{3}}{\partial E_{3}}=\frac{P_{0}}{\varepsilon_{0}}cos\psi \frac{\partial \psi }{\partial E_{3}}=\frac{k_{1}P_{0}}{\varepsilon_{0}}\frac{1}{2sin\psi }$$

where we obtained ∂ *ψ*/ ∂ *E*_3_ = *k*_1_/*sin*2*ψ* from Equation (6). The zero field relative dielectric constant, assuming that polarization rotation determines the dielectric properties of the ferroelectric, results in a relation between the fitting parameters $\varepsilon_{33}^{rot}\left(0\right)=k_{1}P_{0}/2\varepsilon_{0}sin\psi_{0}$. The high field value is $\varepsilon_{33}^{rot}\left(\infty \right)=k_{1}P_{0}/2\varepsilon_{0}$. In Figure A1 the measured and modelled curves for the relative dielectric constant as function of field are shown. At high fields Equation (A1) describes the experimental values $\varepsilon_{33}$ quantitatively fairly well, without any additional fitting. At low fields there are larger deviations from the rotation model, due to the effects of polarization switching. This is discussed in Appendix B below.

## Appendix B – polarization switching

At low fields the *P* – *E* loops (Figure 3(a)) are slightly hysteretic. We expect that in this part of the loop both rotation and switching takes place so that the average polarization can be written as:

(B1) $$P_{3}^{rot+sw}=\phi p_{u}+\left(1-\phi \right)p_{d}=P_{0}\left(2\phi -1\right)\sin\psi$$

$$P_{12}^{rot+sw}=P_{0}\left(2\phi -1\right)\cos\psi$$

Here *ϕ* is the volume fraction of material in which the polarization is in the (+3) direction with the value *p*_*u*_ = *P*_0_ sin *ψ* and *p*_*d*_ = –*P*_0_ sin *ψ* is the value of the polarization in the (–3)-oriented domains. For ϕ=1 (0) all domains have polarization up (down), and for *ϕ* = 1/2 the polarizations in the up and downwards oriented domains cancel each other. The reason for the switching is not that the coercive field is compensated by the applied field, which it is evidently not the case at the high fields at which switching sets in, but that the total energy of the system with polarization up and down domains is lower than that of a (quasi-) monodomain state. A strong analogy with the polarization rotation and switching mechanisms is observed in uniaxial ferromagnetic systems with low anisotropy energy and negligible coercive field [28]. In the magnetic system the polydomain state arises from the reduction of the demagnetization energy term in the total energy. Stephenson and Elder [29] described the appearance of 180° stripe domains in compressively strained PbTiO_3_ thin films, due to reduction of the depolarization energy. In analogy with the model of [28] the polarization switching is between polarization vectors with the same in-plane polarization components but opposite oriented out-of-plane components. It may not only be the reduction in polarization energy that initiates and drives the switching. The results of Kukhar et al. [22] on clamped ferroelectric polydomain PZT films indicate that the ferroelectric *c/a* oriented tetragonal domain formation arises due to a reduction of the overall elastic energy in the film. We assume that a similar mechanism is possible in the PMN-PT films, in which the rhombohedrally distorted monodomain splits up in domains with opposing distortion angles and thus out-of-plane polarization directions, to reduce the overall elastic energy. To simplify the analysis we assume that at low fields the switching process dominates and that the tilt angle of the polarization in the different domains is equal to a value $\psi_{k}$, with plus sign for upwards oriented polarization domains and minus sign for downwards oriented domains. *ψ*_*k*_ is approximately equal to the tilt angle at which polarization rotation changes into switching, occurring at a field *E*_*k*_. Considering only switching Equation (B1) becomes:

(B2) $$P_{3}^{sw}=P_{0}\left(2\phi -1\right)\sin\psi_{k}\;P_{12}^{sw}=P_{0}\left(2\phi -1\right)\cos\psi_{k}$$

The switching branches of the *P* – *E* loops are approximately linear for low field strength, thus one can approximate $\phi =\left(E-E_{c\pm }\right)/2E_{k}+1/2$, for the rising (with $E_{c+})$and falling (with $E_{c-}=-E_{c+})$branches. (Here we removed any self-bias field by shifting the experimental loops along the field axis accordingly.) The rising ‘switching’ branch (index r) cuts the ‘rotation branches’ for (negative) *ψ*_*k*-_ at *E*_*k*-,*r*_ = –*E*_*k*_ + *E*_*c*+_ and (positive) *ψ*_*k*+_ at *E*_*k*+,*r*_ = *E*_*k*_ + *E*_*c*+_. The width of the switching branch is $\Delta E_{k}=E_{k+,r}-E_{k-,r}=2E_{k}$. For the falling branch (index f) *E*_*k*+,*f*_ = *E*_*k*_ + *E*_*c*-_ and *E*_*k*-,*f*_ = - *E*_*k*_ + *E*_*c*-_. In Figures 5(b) and 6(b) the switching branches are indicated by the dashed red lines. We estimate $E_{k}\approx \left(\left|E_{k+,r}\right|+\left|E_{k+,f}\right|\right)/2$ to be 75 kV cm ^– 1^ for both the (001)- and (110)-oriented films. Since we have no model underlying the field dependence of the domain fraction yet, it is not clear what determines this value quantitatively, although in analogy with the model of [28] it is likely to be related to the strength of the polarization easy axis and depolarization energies. With that the polarization of the switching branches can be written as:

(B3) $$P_{3,r/f}^{sw}=\left(\frac{E-E_{c\pm }}{E_{k}}\right)P_{0}\sin\psi_{k}$$

The relative susceptibility due to switching follows as:

(B4) $$\varepsilon_{33,r/f}^{sw}=\frac{P_{0}\sin\psi_{k}}{\varepsilon_{0}E_{k}}$$

and is, in this approximation of constant polarization angle *ψ*_*k*_, constant in the field range $E_{k-,r}<E<E_{k+,r}$ for the rising branch and $E_{k-,f}<E<E_{k+,f}$ for the falling branch. The peaks of the rising and falling branches of $\varepsilon_{33,r/f}^{sw}$ are shifted with respect to each other over *E*_*c*+_ - *E*_*c*-_ = 2*E*_*c*+_.

(Figure A1 shows the measured relative susceptibility *ɛ*_*CV*_ (corrected for the self-bias) from *C* – *V* measurements of the (001)- and (110)-oriented devices, characterized by Figures 5 and 7, and the curves corresponding to the derivative *ɛ*_*PE*_ = ∂ *P*_3_/*ɛ*_0_ ∂ *E*_3_ of the measured polarization loops (after self-bias and leakage correction). There is a large difference between *ɛ*_*CV*_ and *ɛ*_*PE*_ at low field bias, the latter being about 1.5× larger, whereas at high fields *ɛ*_*CV*_ and *ɛ*_*PE*_ are approximately equal. The susceptibility *ɛ*_33_(*E*) calculated from Equation (A1) with the same fitting parameter values as used for Figure 5(a) and (b), valid for the rotation part of the polarization branch, reproduces the measured high field susceptibility quite accurately, as well as the maximum at zero field. At low fields one would expect a contribution from the switching to the susceptibility and *ɛ*_33_(*E*) is estimated from Equation (B4). It reproduces the low-field values of the experimental *ɛ*_*PE*_(*E*) fairly well, as well as the peak separation in *ɛ*_*PE*_(*E*), but overestimates the measured maximum *ɛ*_*CV*_ significantly. On the other hand the peak width predicted by rotation polarization only is much smaller than the observed width, which is better reproduced by the switching model. Using *ψ*_0_ instead of *ψ*_*k*_ in Equation (B4) gives a maximum value of the switching susceptibility that is more comparable to that of the measured *ɛ*_*CV*_-loop. These are indications that polarization rotation still takes place in the switching part of the loop, causing the reduced peak height. However the very small separation (and unequal heights) of the two peaks in *ɛ*_*CV*_ cannot be explained by the simple switching model, given above. We think that the negligible peak separation in the *ɛ*_*CV*_ curves is related to the inertial and viscous forces acting on the moving domain walls (DWs). The *P* – *E* loop and therefore *ɛ*_*PE*_ are measured with a field bias changing at a constant rate, $E\left(t\right)=at$. The DWs, present in the switching part of the *P* – *E* loop, therefore move at a constant speed determined by the change in domain fraction and not subject to inertial forces, although they may lag behind the change in the field, due to the viscous forces. This explains why the *ɛ*_*PE*_ is estimated better by the switching model at low fields and by slow rotation of the polarization at high fields where there are no DWs anymore. The *C* – *V* loop on the other hand is measured with a field bias of the form $E\left(t\right)=at+E_{ac}sin\omega t$. The polarization response on the oscillatory field is measured with a lock-in amplifier, supplying the in-phase signal, proportional to *ɛ*_*CV*_ and the out-of-phase signal from which the loss tangent *tanδ*_*CV*_ is obtained. This method therefore measures the oscillation frequency (and amplitude) dependent response of the domain-wall movement, $\partial \phi /\partial E=f\left(\omega ,E_{ac},\psi \right)$, as well as the polarization rotation within the domains. Considering that the *C* – *V* curve is quantitatively fairly well described by Equation (A1), taking only polarization rotation into account, suggests that rotation is the dominant mechanism determining *ɛ*_*CV*_. The broadening of the zero-field peak may then be ascribed to the additional contribution from domain wall motion to *ɛ*_*CV*_. The high losses measured in the switching regime indeed indicate that the DW displacement is subject to a substantial loss mechanism and inertial reaction of the DW self-mass [30].

Considering only polarization rotation *d*_33_ is given by $d_{33}^{rot}$, producing a square *d*_33_ – *E* loop: for the falling branch $d_{33,f}\left(E>-\left|E_{3\text{min}}\right|\right)=P_{0}^{2}\left(c-b\right)k_{1}$ and for the rising branch $d_{33,r}\left(E<\left|E_{3\text{min}}\right|\right)=-P_{0}^{2}\left(c-b\right)k_{1}$. These curves are shown in Figure 5(a) (blue lines) together with the experimental loop (shifted over the self-bias field). If only switching causes the change in the polarization in the ‘switching’ branch, then the unit cell dimensions do not change, hence *S*_3*u*_ = *S*_3*d*_ due to the quadratic dependence of *S*_3*u*/*d*_ on the polarization components. *S*_3*u*/*d*_ is the strain in the upward/downward polarized domain. The average strain $S_{3}=\phi S_{3u}+\left(1-\phi \right)S_{3d}=aS_{m}+\left(bcos^{2}\psi_{k}+csin^{2}\psi_{k}\right)P_{0}^{2}$ is then constant and ∂ *S*_3_/ ∂ *E*_3_ = 0. Hence the pure switching model predicts that the piezoelectric coefficient in a regime where only switching takes place is zero in the field ranges $E_{k-,r/f}<E<E_{k+,r/f}$ for the rising (falling) branch (red dashed lines in Figures 5(a) and 7(a)). If both switching and rotation are contributing then ∂ *S*_3_/ ∂ *E*_3_ again becomes equal to Equation (5). The experimental *d*_33_ – *E* data in the switching regime show a steep slope over the approximate field range in which switching takes place $\left\{-E_{k}\mp E_{c}^{\pm },E_{k}\pm E_{c}^{\pm }\right\}$. Considering that the displacement is measured by a lock-in technique using a drive field bias of the form $E\left(t\right)=at+E_{ac}sin\omega t$, it is expected that, similarly as for the *ɛ*_*CV*_ measurements, there is a significant effect of the lossy and inertial DW motion in the switching range. The analysis indicates that field driven polarization rotation also takes place in the switching branches. Hence the simple model using either switching or rotation should incorporate the polarization rotation in the switching branch. Further the analysis indicates that the finite self-mass and viscous motion of the DWs influence the low-field susceptibility peaks and the piezoelectric constant in the switching regime.
